# Associations of heart failure to prevalence of haematologic- and solid malignancies in southern Sweden: A cross-sectional study

**DOI:** 10.1371/journal.pone.0292853

**Published:** 2023-10-13

**Authors:** Mia Scholten, Anders Halling

**Affiliations:** Department of Clinical Sciences, Malmö, Lund University, Malmö, Sweden; Royal College of Surgeons in Ireland, IRELAND

## Abstract

**Background:**

Heart failure (HF) and cancer are common diseases among the elderly population. Many chronic diseases, including diabetes mellitus (DM), share risk factors and increase the incidence of HF and cancer. The aim of this study was to investigate if there was an association between HF and the prevalence of haematologic- and solid malignancies.

**Methods:**

The study population was comprised of almost one million adults living in southern Sweden in 2015. All participants were divided into seven age groups from 20 and onwards, and 10 percentiles according to their socioeconomic status (SES). All data concerning diagnoses from each consultation in both primary- and secondary health care were collected during 18 months. The prevalence of haematologic and solid malignancies was measured separately for men and women, age groups, SES and multimorbidity levels. Multivariable logistic regression was used to determine the associations between HF and the probability of having haematologic- and solid malignancies in more complex models including stratifying variables.

**Results:**

People with HF had a higher prevalence of haematologic- and solid malignancies than the general population, but a lower prevalence of solid malignancies than the multimorbid population. The people with HF had an increased OR for haematologic malignancies, 1.69 (95% CI 1.51–1.90), and solid malignancies, OR 1.21 (95% CI 1.16–1.26), when adjusted for gender and age. In more complex multivariate models, multimorbidity explained the increased OR for haematologic- and solid malignancies in people with HF. Increasing socioeconomic deprivation was associated with a decreased risk for solid malignancies, with the lowest risk in the most socioeconomically deprived CNI-percentile.

**Conclusions:**

HF was shown to be associated with malignancies, especially haematologic malignancies. Multimorbidity, however, was an even more important factor for both haematologic- and solid malignancies than HF in our study, but not socioeconomic deprivation. Further research on the interactions between the chronic conditions in people with HF is warranted to examine the strength of association between HF and malignancies.

## Introduction

HF and cancer are common diseases among the elderly population. Many chronic diseases, including DM, share risk factors such as sedentary lifestyle, genetics, obesity and tobacco smoking, which are also known risk factors for HF and cancer [1–3]. HF is mostly a complication or end stage of other cardiovascular diseases (CVD) and is strongly associated with multimorbidity. Results from our previous study of this population have shown that almost all (99.07%) adult people with HF had multimorbidity [4]. Furthermore, patients in the most socioeconomically deprived percentile were affected by HF many years earlier than patients in the more socioeconomically affluent percentiles [4]. People with HF due to ischaemic heart disease have been reported to have a higher incidence of cancer compared to HF due to other aetiologies [5]. Chronic diseases such as hypertension and DM have also been shown to be associated with an increased risk for cancer [6,7].

Cancer and CVD are both associated with a high mortality rate. Around 15% of the global death burden is due to cancer and 30% is a result of CVD [8]. Both HF and cancer have been linked to increased chronic inflammation and oxidative stress, which play central roles in the pathophysiology of both diseases [2]. Several studies have provided evidence that the progression of either HF or cancer is linked to enhanced tissue inflammation [9,10]. In addition, it has been speculated that radiation, epigenetic mechanisms and regenerative signalling are all potential links to both HF and cancer [11]. RAAS activation—a common response to HF—has been shown to be strongly associated with an increase in tumour angiogenesis, angiogenetic factor expression, invasiveness and metastasis leading to a poor prognosis [12]. Treatment of HF with angiotensin receptor blockers (ARBs) or ACE inhibitors (ACEi) has been hypothesised to reduce the risk for cancer based on data derived from both the SOLVD and CHARM studies [13]. A Danish study enrolling 9,307 people with HF (predominantly with left ventricular ejection fraction < 45%) showed a higher incidence rate of malignancies compared to the general population [14].

DM, which is a common comorbidity in people with HF, represents a risk factor for cancer, particularly hepatocellular, hepatobiliary, pancreas, breast, ovarian, endometrial, and gastrointestinal cancers [15]. Between 8% and 18% of individuals with diagnosed cancer also have DM as a comorbidity [15]. Moreover, there is evidence showing that DM is associated with higher cancer mortality [16,17]. Although the links between DM and cancer are not yet completely understood, biological mechanisms such as increased bioactivity of insulin-like growth factor 1, chronic inflammation, oxidative stress, dysregulations of sex hormones, direct effects of excess glucose and insulin signalling are most likely involved [18–21].

In this study, we aimed to investigate if there was an association between HF and the prevalence of haematologic- and solid malignancies. DM, as a common comorbidity in people with HF, was also analysed to study its impact on the association between HF and the probability of having haematologic- and solid malignancies.

## Materials and methods

### Setting and study population

Scania is the southernmost county of Sweden and had approximately 1.3 million inhabitants in 2015 [22]. The biggest city in Scania is Malmö, which had about 320,000 inhabitants during the study period, and is ranked as the third largest city in Sweden [22]. About a third of the residents in Malmö were born abroad with most countries in the world being represented [23], whilst approximately 25% of the whole study population were born abroad [24]. Almost half of the inhabitants (48.40%) in Malmö were under 35 years of age in 2015 [25].

This is a cross-sectional study, which included all inhabitants from the age of 20 and older living in Scania during the last week of 2015. This age cut-off was chosen because children and younger people tend to have subtypes of HF with other aetiologies than those found in older adults. The study population was divided into seven age groups, ranging from 20 to 80. The age group 20 included all individuals from 20 to 29 years, and the age group 50 included all individuals from 50 to 59 years, and so on. The age group 80 included all individuals aged 80 and older. The general population was comprised of all the participants in our study.

### Data source and measurements

Most residents in Sweden are listed at a primary health care centre (PHC). A total of 152 PHCs were in operation during 2015 in Scania, with an average of 8587 listed patients (95% CI 7971–9293) at each PHC. The data we used in this study were retrieved from the Scania County Council health care register that contains anonymised registry information from 981,383 (about a tenth of the Swedish population) inhabitants, including age, gender, socioeconomic status and diagnostic data. **This database has a good quality because all patient data are included from both private and public health care, which is also a part of the Swedish national patient register.** During a period of 18 months (July 2014—December 2015), we collected all data concerning diagnoses from each consultation in both primary- and secondary health care. Diagnoses were recorded according to the International Statistical Classification of Diseases and Related Health Problems version 10 (ICD 10) (Appendix Table 1 in S1 Appendix).

### Socioeconomics

We used the term Care Need Index (CNI) [26] to divide the PHCs into 10 percentiles depending on their socioeconomic status. CNI is based on different measures of a group, which in this case was the patients listed at different PHCs in Scania. CNI 1 was assigned to those patients listed at PHCs that belonged to the most socioeconomically affluent percentile, and CNI 10 was assigned to those patients listed at PHCs that belonged to the most socioeconomically deprived percentile [26].

### Multimorbidity

Multimorbidity is defined as the simultaneous coexistence of two or more chronic conditions in the same person [27]. To measure multimorbidity, we used a method to identify chronic conditions developed by A Calderòn-Larrañaga *et al*. at the Aging Research Centre in Stockholm [27]. They analysed the full list of ICD-10 codes on a four-digit level to define if a diagnosis is chronic or not in an elderly population. To determine if a condition is chronic or not the following key features were identified and discussed concerning their pertinence and suitability in older populations: duration, course, reversibility, treatment, and consequences. They were then grouped into 60 chronic disease categories [27]. We applied their definition and list of chronic conditions to estimate multimorbidity in our study population. Multimorbidity was then calculated by counting the number of chronic conditions in each patient. To study the degree of multimorbidity, the patients were further divided into groups MM0 (less than two chronic conditions), MM1 (two to four chronic conditions), MM2 (five to nine chronic conditions) and MM3 (10 chronic conditions or more).

### Statistical analyses

The study population was divided into HF-, DM patients and the general population with and without haematologic- respective solid malignancies, which were further stratified according to gender, age and multimorbidity level. We used frequencies, percentages and cross-tabulations for descriptive analysis and Chi-square-test to calculate the p-values. A p-value ≤ 0.05 was considered to be statistically significant. A p-value between 0.01 and 0.05 was considered to be a low level of significance; between 0.001 and 0.01 as a middle level of significance; and ≤ 0.001 as a high level of significance. Multivariate logistic regression of increasing complexity (Models A—E) was used to analyse the associations between HF and the probability of having haematologic-respective solid malignancies. The different variables in Model A—E are listed in Appendix Table 2 in S1 Appendix. All statistical analyses were performed with STATA version 17.0 (Stata Corporation, Texas, USA).

### Ethics

Data in the present study are based on anonymised information provided by the Scania County Council. They provided anonymised information for research purposes once the study had been approved by the Ethics Committee at Lund University. We confirm that all analyses of the human data were performed in accordance with relevant guidelines and regulations as stated in the Declaration of Helsinki.

The study participants were not involved in the recruitment to the study by themselves. Due to the requirement concerning anonymised data, each individual could not be asked for consent to participate; active refusal of participation was instead applied. This was done by publishing information about the planned study in the Swedish local newspaper “Sydsvenskan”. The advertisement outlined the study and contained information on how to contact the research manager (first author) to opt out of the study. The regional Ethical Review Board at Lund University approved the study (application no. 2018/778), which deemed the informed consent process as unnecessary, since the study results are published anonymised on a group level, and cannot be traced to every study participant.

## Results

The total prevalence of HF in the study population was 2.06%, of whom 28.04% also had DM. A total of 0.39% of the study population had haematologic malignancies and 4.97% had solid malignancies (Table 1). The prevalence of haematologic malignancies in people with HF was 1.73% and for solid malignancies, it was 16.60%. The total prevalence of DM was 6.50% in the study population, but the prevalence of haematologic- (0.83%) and solid malignancies (10.47%) was lower compared to the people with HF (Table 1). The people with HF having 2–9 chronic conditions had a higher prevalence of haematologic malignancies compared to the general population, but not the people with HF having 10 comorbidities or more. People with HF had a lower prevalence of solid malignancies than the general population with multimorbidity of all levels (Table 1).

**Table 1:** Prevalence of haematologic- and solid malignancies in the general population compared to patients with heart failure or diabetes mellitus.

	General population					Haematologic malignancies						Solid malignancies					
		Heart failure		Diabetes mellitus		General population		Heart failure		Diabetes mellitus		General population		Heart failure		Diabetes mellitus	
	N	%	N	%	N	%	N	%	N	%	N	%	N	%	N	%	N
**Gender**																	
men	482355	2.19	10563	7.57	36516	0.43	2055	1.92	203	0.94	345	5.13	24757	19.41	2051	11.60	4236
women	499028	1.93	9630	5.45	27210	0.36	1802	1.52	146	0.67	181	4.61	23018	13.51	1301	8.95	2436
p-value*									NS		< 0.001				< 0.001		< 0.001
**Age (years)**																	
20–29	180876	0.03	56	0.76	1373	0	0	0	0	0	0	0.45	818	0	0	0.51	7
30–39	170080	0.06	106	1.28	2175	0.09	152	0.94	1	0.23	5	0.80	1367	1.89	2	1.47	32
40–49	175095	0.22	382	3.02	5288	0.15	268	1.05	4	0.26	14	1.59	2783	3.14	12	2.29	121
50–59	152432	0.69	1048	6.94	10586	0.29	445	1.43	15	0.46	49	3.56	5422	5.92	62	4.28	453
60–69	149540	2.13	3184	12.52	18718	0.68	1021	1.79	57	0.74	138	8.67	12961	11.78	375	9.41	1762
70–79	98599	6.02	5938	17.28	17037	1.15	1136	1.92	114	1.20	204	15.00	14793	17.75	1054	16.06	2736
≥ 80	54761	17.31	9479	15.61	8549	1.33	728	1.67	158	1.36	116	17.59	9631	19.49	1847	18.26	1561
p-value*									< 0.001		< 0.001				< 0.001		< 0.001
**Multimorbidity level** **																	
0–1	604221	0.03	188	0.53	3190	0.07	451	0	0	0	0	1.03	6218	0	0	0	0
2–4	260764	1.49	3875	9.51	24788	0.58	1502	0.75	29	0.33	82	7.73	20165	6.37	247	4.84	1199
5–9	105241	11.16	11748	28.95	30463	1.52	1603	1.73	203	1.02	312	17.49	18410	17.08	2007	13.85	4218
≥ 10	11156	39.28	4382	47.37	5285	2.70	301	2.67	117	2.50	132	26.73	2982	25.06	1098	23.75	1255
p-value*									< 0.001		< 0.001				< 0.001		< 0.001

*p-value of proportion of haematologic- and solid malignancies according to heart failure or diabetes mellitus.

**Multimorbidity level = number of chronic conditions.

N = total number of individuals; NS = Not significant, P > 0.05.

People with HF had an increased OR for haematologic malignancies, OR 1.69 (95% CI 1.51–1.90), and solid malignancies, OR 1.21 (95% CI 1.16–1.26) when adjusted for age and gender (Model B, Tables 2 and 3). These ORs decreased slightly when further adjusted for DM. DM had no significance for the probability of having haematologic malignancies, but an increased probability for solid malignancies, OR 1.07 (95% CI 1.04–1.10), when adjusted for HF, age and gender (Model C, Tables 2 and 3). The ORs of HF and DM did not change significantly when further adjusted for SES. Increasing socioeconomic deprivation was associated with a decreased risk for solid malignancies. The most socioeconomically deprived CNI percentile had a 35% lower risk for solid malignancies than the most socioeconomically privileged CNI-percentile (Model D, Tables 2 and 3). When multimorbidity was added in Model E, the ORs of HF and DM disappeared for both haematologic- and solid malignancies (Model E, Tables 2 and 3).

**Table 2:** Odds ratios (OR) and 95% confidence intervals (CI) for haematologic malignancies in different categories.

	Model A	Model B	Model C	Model D	Model E
	OR (95% CI)	OR (95% CI)	OR (95% CI)	OR (95% CI)	OR (95% CI)
**Gender**					
men	1.31 (1.23–1.40)	1.30 (1.22–1.38)	1.30 (1.22–1.38)	1.30 (1.21–1.38)	1.45 (1.36–1.54)
women	1	1	1	1	1
**Age (years)**					
20–29	1	1	1	1	1
30–39	1.51 (1.18–1.93)	1.51 (1.18–1.93)	1.51 (1.18–1.93)	1.50 (1.17–1.92)	1.32 (1.03–1.69)
40–49	2.59 (2.07–3.23)	2.58 (2.06–3.23)	2.58 (2.06–3.23)	2.54 (2.03–3.18)	1.86 (1.49–2.33)
50–59	4.94 (4.00–6.11)	4.92 (3.98–6.08)	4.91 (3.97–6.06)	4.85 (3.93–5.99)	2.66 (2.14–3.29)
60–69	11.64 (9.54–14.21)	11.47 (9.39–14.00)	11.41 (9.34–13.93)	11.22 (9.18–13.71)	4.46 (3.63–5.47)
70–79	19.86 (16.28–24.21)	19.04 (15.61–23.23)	18.91 (15.49–23.08)	18.59 (15.23–22.71)	5.38 (4.37–6.62)
≥ 80	23.61 (19.26–28.93)	21.04 (17.13–25.85)	20.93 (17.03–25.72)	20.69 (16.83–25.43)	5.07 (4.09–6.28)
**Heart failure**		1.69 (1.51–1.90)	1.68 (1.50–1.89)	1.69 (1.50–1.90)	0.94 (0.83–1.06)
**Diabetes mellitus**			1.04 (0.95–1.15)	1.05 (0.95–1.15)	0.56 (0.51–0.61)
**Socioeconomic percentile**					
1 (highest)				1	1
2				0.97 (0.85–1.10)	1.01 (0.89–1.15)
3				0.99 (0.87–1.13)	1.00 (0.88–1.14)
4				0.91 (0.79–1.04)	0.91 (0.79–1.04)
5				0.93 (0.81–1.08)	0.95 (0.82–1.09)
6				0.88 (0.77–1.00)	0.91 (0.80–1.04)
7				0.94 (0.82–1.08)	0.99 (0.86–1.13)
8				0.93 (0.82–1.06)	0.97 (0.85–1.10)
9				0.82 (0.72–0.95)	0.89 (0.78–1.03)
10 (lowest)				0.86 (0.74–1.00)	0.94 (0.80–1.09)
**Multimorbidity level** *					
0–1					1
2–4					5.44 (4.87–6.07)
5–9					11.95 (10.62–13.46)
≥ 10					21.41 (18.10–25.33)

*Multimorbidity level = number of chronic conditions.

**Table 3:** Odds ratios (OR) and 95% confidence intervals (CI) for solid malignancies in different categories.

	Model A	Model B	Model C	Model D	Model E
	OR (95% CI)	OR (95% CI)	OR (95% CI)	OR (95% CI)	OR (95% CI)
**Gender**					
men	1.28 (1.26–1.31)	1.28 (1.25–1.30)	1.27 (1.25–1.30)	1.27 (1.25–1.30)	1.42 (1.39–1.45)
women	1	1	1	1	1
**Age (years)**					
20–29	1	1	1	1	1
30–39	1.78 (1.63–1.94)	1.78 (1.63–1.94)	1.78 (1.63–1.94)	1.76 (1.61–1.92)	1.58 (1.44–1.72)
40–49	3.55 (3.28–3.84)	3.55 (3.28–3.84)	3.54 (3.28–3.83)	3.43 (3.18–3.71)	2.64 (2.44–2.86)
50–59	8.12 (7.54–8.74)	8.11 (7.53–8.73)	8.07 (7.50–8.69)	7.85 (7.29–8.45)	4.73 (4.39–5.10)
60–69	20.96 (19.52–22.50)	20.86 (19.43–22.40)	20.69 (19.27–22.22)	19.93 (18.56–21.40)	9.20 (8.55–9.89)
70–79	39.23 (36.55–42.11)	38.75 (36.10–41.60)	38.31 (35.69–41.14)	36.85 (34.31–9.57)	12.91 (12.00–13.88)
≥ 80	48.18 (45.29–52.32)	47.01 (43.72–50.56)	46.58 (43.31–50.09)	45.20 (42.03–48.62)	13.48 (12.51–14.53)
**Heart failure**		1.21 (1.16–1.26)	1.20 (1.15–1.24)	1.20 (1.16–1.25)	0.69 (0.66–0.72)
**Diabetes mellitus**			1.07 (1.04–1.10)	1.08 (1.05–1.11)	0.58 (0.57–0.60)
**Socioeconomic percentile**					
1 (highest)				1	1
2				0.94 (0.91–0.97)	0.98 (0.94–1.01)
3				0.85 (0.82–0.89)	0.86 (0.82–0.89)
4				0.86 (0.83–0.90)	0.86 (0.83–0.90)
5				0.85 (0.81–0.89)	0.86 (0.82–0.90)
6				0.83 (0.80–0.86)	0.85 (0.81–0.88)
7				0.88 (0.84–0.91)	0.91 (0.88–0.95)
8				0.86 (0.83–0.90)	0.89 (0.85–0.92)
9				0.77 (0.74–0.81)	0.83 (0.80–0.87)
10 (lowest)				0.65 (0.62–0.69)	0.70 (0.67–0.74)
Multimorbidity level*					
0–1					1
2–4					4.78 (4.64–4.93)
5–9					9.13 (8.83–9.44)
≥ 10					16.04 (15.18–16.95)

The ORs for haematologic malignancies exceeded the ORs for solid malignancies in the multimorbid patients of all levels (Model E, Tables 2 and 3). The probability for both haematologic- and solid malignancies increased with advancing age, but multimorbidity had a higher significance for the probability of haematologic malignancies than all age groups (Model E, Table 2). Meanwhile, only the multimorbid patients with 10 chronic conditions or more, including HF and DM, had a higher significance for the probability of solid malignancies than all age groups (Model E, Table 3). Men had a higher OR for both haematologic- and solid malignancies than women, which increased further when adjusted for multimorbidity (Tables 2 and 3).

## Discussion

The total prevalence of HF was 2.06% and for DM it was 6.50% in the study population. Both conditions had a higher prevalence of haematologic- and solid malignancies than the general population, but a lower prevalence of solid malignancies than the multimorbid population. Although the total prevalence of DM was approximately three times higher than HF, the DM patients had a lower prevalence of both haematologic- and solid malignancies than the people with HF. In spite of the high prevalence of DM in people with HF (28.04%), they had only slightly decreased ORs when further adjusted for DM. When multimorbidity was adjusted together with HF and DM, the ORs of HF and DM disappeared for both haematologic- and solid malignancies. These results highlight the strong association between multimorbidity and malignancies contributed by both HF and DM in our study. Nevertheless, only these two conditions together did not explain the increased probability for haematologic- or solid malignancies in the MM1 group as the ORs were 5.44 (95% CI 4.87–6.07) and 4.78 (95% CI 4.64–4.93), respectively. These findings indicate that both HF and DM contributed to the increased probability of haematologic and solid malignancies but, in combination with other chronic conditions, might be even stronger risk factors.

People with HF had a higher probability for haematologic than solid malignancies when adjusted for age and gender, thus suggesting a stronger association between HF and haematologic malignancies than solid malignancies. Even multimorbidity of all levels, including HF and DM, had higher ORs for haematologic than solid malignancies, indicating that multimorbidity was a more important factor for haematologic malignancies in our study. The more socioeconomically deprived CNI percentiles had a significantly lower risk for solid malignancies than the most socioeconomic privileged CNI-percentile. These findings could be explained by a lower adherence to screening guidelines in the socioeconomically deprived population resulting in a later diagnosis [28,29].

When stratifying for multimorbidity levels, only the people with HF having 2–9 chronic conditions had a higher prevalence of haematologic malignancies compared to the general population, but not the people with HF having 10 comorbidities or more. A previous study reported a lower prevalence of people with HF having10 chronic conditions or more than those belonging to the MM2 group [4], who presumably developed fewer malignancies due to their high mortality rate.

The multimorbid patients, including HF and DM, had a higher OR for haematologic malignancies than all age groups. For solid malignancies, only the multimorbid patients with 10 chronic conditions or more had a higher OR than all age groups. These findings indicate that multimorbidity was a more important factor for haematologic malignancies independent of age, meanwhile multimorbidity with 10 chronic conditions or more, including HF and DM, was a more important factor for solid malignancies independent of age.

People with HF are characterised by reduced overall perfusion and chronic inflammation, which could contribute to cancer development [2]. On the other hand, some specific mutations in haematologic malignancies could also cause HF development [30]. Another plausible explanation is HF in these patients may constitute a complication of cancer treatment [31,32]. A retrospective study revealed that the probability of incurring doxorubicin-induced HF was related to the total dose of doxorubicin administered: the higher the cumulative amount of administered drug, the more increased risk for HF. An increase in drug-related HF was also observed with advancing patient age, independent of performance status, gender, race, and tumour type [32].

A meta-analysis including around four million DM patients and 10,516 leukaemia patients reported a 3.48-fold relative risk of leukaemia within the first year of type 2 DM diagnosis than the population with normal glucose values. The incidence of leukaemia was also significantly increased in patients with type 2 DM for 1–10 years. After 10 years of the DM diagnosis, the relative risk of leukaemia declined to the level of the general population among these patients [33]. These findings could be explained by a normalisation of fasting serum glucose levels in the DM patients by anti-diabetic treatment many years after diagnosis, which prevents the development of hyperglycaemia-induced leukaemia [34]. A comprehensive meta-analysis regarding type 2 DM and the risk of developing cancer has shown that the presence of DM is associated with a 10% increase in the relative risk (RR) of developing cancer (RR, 1.10; 95% CI 1.04–1.17) [20], and the hazard ratio for cancer incidence was 1.76, (95% CI 1.71–1.81, P < 0.001), for people with HF [35]. These results are comparable with the DM patients in our study, who had no significance for haematologic malignancies, but an increased probability for solid malignancies, although less than the people with HF.

In a prospective cohort study, which included almost 300,000 participants from seven European countries, middle-aged and free of cancer, CVD, and DM [1]. Cox regression was used to calculate hazard ratios of developing multimorbidity in relation to body mass index (BMI), smoking status, physical activity, alcohol intake, adherence to the Mediterranean diet, and their combination as a healthy lifestyle index score. During a median follow-up of 11 years, 1.11% of the participants developed multimorbidity including cancer and cardiometabolic diseases [1]. A healthy lifestyle was inversely associated with multimorbidity. After a diagnosis of DM, the 10-year risks of multimorbidity were 30% for men and 18% for women with healthy lifestyles. For unhealthy lifestyles, the figures were 40% for men and 25% for women, respectively. After a diagnosis of CVD, the 10-year risks of multimorbidity were slightly lower than the DM patients but were comparatively higher than the cancer patients [1]. The importance of these common risk factors for lifestyle-related multimorbidity could, in part, explain the results of our study.

Another cross-sectional study reported a prevalence of 9% having any type of cancer and 38% multimorbidity [36]. Respiratory conditions and arthritis were statistically significantly associated with having all-site cancer, with OR 1.3 (95% CI 1.1–1.6) and OR 1.5 (95% CI 1.2–1.8), respectively. Multimorbidity was statistically significantly associated with having all-site cancer (OR 1.4, 95% CI 1.2–1.7), cervical cancer (OR 2.6, 95% CI 1.2–5.4), and bladder cancer (OR 2.8, 95% CI 1.0–7.6) [36]. These results suggest that various cancer types are differently associated with various chronic conditions, and most likely composition of multimorbidity, which could explain the substantial increase of OR with advancing multimorbidity level in our study.

The estimated cancer deaths are expected to overcome those for ischaemic heart disease, with a 2.08-fold increase (1.76-fold for the increase in ischaemic heart disease) by the year 2060. Thus, cancer will become the leading cause of mortality globally immediately after 2030 [37]. Prevention of cancer is therefore a pressing issue. Our study revealed new factors associated withmalignancies, but the pathophysiology in these patient groups is still elusive and has to be explored in further studies.

### Strengths and limitations

We have analysed the difference in the prevalence of haematologic- and solid malignancies between the general population and patients with two common chronic diseases. The difference in prevalence between the general population and these patient groups had a high level of statistical significance in all age groups and multimorbidity levels. We used multivariable logistic regression to compare the ORs of different variables in relation to each other to determine their significance for haematologic- and solid malignancies. Our findings have similarities with corresponding studies in other countries, which improves the validity and credibility of this study. The study population was heterogeneous comprising adults from many nationalities, which reduces the probability of consanguinity and its consequences for health.

We had no data on the hereditary forms of common solid malignancies, like breast cancer, colorectal cancer and prostate cancer. Data on heredity in our study could even influence the variables gender, age, HF, DM and multimorbidity level, but not socioeconomic status. We had no data on the subtypes of HF, which have different impacts on the complications and outcomes for these patients. The data on comorbidities in the people with HF were lacking as well, which might have stronger associations with haematologic- or solid malignancies than HF. Many haematologic- and solid malignancies affect individuals under 20 years of age more frequently than elderly individuals, which could influence our statistics radically if they were included. Some malignancies are asymptomatic or cause non-specific symptoms during the first year, which usually result in a diagnosis delay. According to current diagnosis rules, only the cancer patients receiving treatments were assigned the diagnosis categories C or D. Thus, cancer patients without current treatments might be underdiagnosed, for example, those with low malignancy or end-stage cancer. The treatments of the examined people with HF and DM could be suboptimal and result in a worse outcome and earlier complications. This was a cross-sectional study, which could have divergent results than a cohort study. Our results are largely based on administrative data and the strength of the association between HF and malignancies remains unclear.

## Conclusions

HF was shown to be associated with malignancies, especially haematologic malignancies. DM explained only a small part of the increased probability for solid malignancies in the people with HF, but contributed to the increased probability for haematologic- and solid malignancies in the multimorbid population together with HF. Multimorbidity was a more important factor for both haematologic- and solid malignancies than HF in our study, but not socioeconomic deprivation. We hypothesise that interactions between chronic conditions are stronger risk factors for malignancies than individual diseases in people with multimorbidity. As most people with HF have multimorbidity, further research is warranted to examine the strength of the association between HF and malignancies.

## Supporting information

S1 Checklist STROBE statement—Checklist of items that should be included in reports of observational studies. (DOCX)

S1 Appendix Appendix—contains the following: Appendix Table 1. ICD-codes for the diseases included in present study. Appendix Table 2. Different variables in Model A—E.
(DOCX)

## References

[1] Freisling H, Viallon V, Lennon H, Bagnardi V, Ricci C, Butterworth AS, et al: Lifestyle factors and risk of multimorbidity of cancer and cardiometabolic diseases: a multinational cohort study. *BMC Med* 2020, 18(1):5. doi: 10.1186/s12916-019-1474-7 31918762 PMC6953215

[2] Koene RJ, Prizment AE, Blaes A, Konety SH: Shared Risk Factors in Cardiovascular Disease and Cancer. *Circulation* 2016, 133(11):1104–1114. doi: 10.1161/CIRCULATIONAHA.115.020406 26976915 PMC4800750

[3] Cignarelli A, Genchi VA, Caruso I, Natalicchio A, Perrini S, Laviola L, et al: Diabetes and cancer: Pathophysiological fundamentals of a ’dangerous affair’. *Diabetes Res Clin Pract* 2018, 143:378–388. doi: 10.1016/j.diabres.2018.04.002 29679627

[4] Scholten M, Midlöv P, Halling A: Disparities in prevalence of heart failure according to age, multimorbidity level and socioeconomic status in southern Sweden: a cross-sectional study. *BMJ Open* 2022, 12(3):e051997. doi: 10.1136/bmjopen-2021-051997 35351700 PMC8966525

[5] Hasin T, Gerber Y, Weston SA, Jiang R, Killian JM, Manemann SM, et al: Heart Failure After Myocardial Infarction Is Associated With Increased Risk of Cancer. *J Am Coll Cardiol* 2016, 68(3):265–271. 27417004 10.1016/j.jacc.2016.04.053 PMC4947209

[6] Stocks T, Van Hemelrijck M, Manjer J, Bjørge T, Ulmer H, Hallmans G, et al: Blood pressure and risk of cancer incidence and mortality in the Metabolic Syndrome and Cancer Project. *Hypertension* 2012, 59(4):802810. doi: 10.1161/HYPERTENSIONAHA.111.189258 22353615

[7] Ballotari P, Vicentini M, Manicardi V, Gallo M, Chiatamone Ranieri S, Greci M, et al: Diabetes and risk of cancer incidence: results from a population-based cohort study in northern Italy. *BMC Cancer* 2017, 17(1):703. doi: 10.1186/s12885-017-3696-4 29070034 PMC5657107

[8] World Health organisation (WHO) NLM classification: WT 500. Geneva, Switzerland: 2014. Global status report on noncommunicable diseases. [http://apps.who.int/iris/bitstream/10665/148114/1/9789241564854_eng.pdf?ua=1].

[9] Anker SD, von Haehling S: Inflammatory mediators in chronic heart failure: an overview. *Heart* 2004, 90(4):464–470. doi: 10.1136/hrt.2002.007005 15020532 PMC1768165

[10] Coussens LM, Werb Z: Inflammation and cancer. *Nature* 2002, 420(6917):860–867. doi: 10.1038/nature01322 12490959 PMC2803035

[11] Hasin T, Iakobishvili Z, Weisz G: Associated Risk of Malignancy in Patients with Cardiovascular Disease: Evidence and Possible Mechanism. *Am J Med* 2017, 130(7):780–785. doi: 10.1016/j.amjmed.2017.02.024 28344133

[12] George AJ, Thomas WG, Hannan RD: The renin-angiotensin system and cancer: old dog, new tricks. *Nat Rev Cancer* 2010, 10(11):745–759. doi: 10.1038/nrc2945 20966920

[13] Cuomo A, Pirozzi F, Attanasio U, Franco R, Elia F, De Rosa E, et al: Cancer Risk in the Heart Failure Population: Epidemiology, Mechanisms, and Clinical Implications. *Curr Oncol Rep* 2020, 23(1):7. doi: 10.1007/s11912-020-00990-z 33263821 PMC7716920

[14] Banke A, Schou M, Videbaek L, Møller JE, Torp-Pedersen C, Gustafsson F, et al: Incidence of cancer in patients with chronic heart failure: a long-term follow-up study. *Eur J Heart Fail* 2016, 18(3):260–266. doi: 10.1002/ejhf.472 26751260

[15] Suh S, Kim KW: Diabetes and Cancer: Cancer Should Be Screened in Routine Diabetes Assessment. *Diabetes Metab J* 2019, 43(6):733–743. doi: 10.4093/dmj.2019.0177 31902143 PMC6943263

[16] Barone BB, Yeh HC, Snyder CF, Peairs KS, Stein KB, Derr RL, et al: Long-term all-cause mortality in cancer patients with preexisting diabetes mellitus: a systematic review and meta-analysis. *Jama* 2008, 300(23):2754–2764. doi: 10.1001/jama.2008.824 19088353 PMC3093051

[17] Campbell PT, Newton CC, Patel AV, Jacobs EJ, Gapstur SM: Diabetes and cause-specific mortality in a prospective cohort of one million U.S. adults. *Diabetes Care* 2012, 35(9):1835–1844. doi: 10.2337/dc12-0002 22699290 PMC3425000

[18] Giovannucci E, Harlan DM, Archer MC, Bergenstal RM, Gapstur SM, Habel LA, et al: Diabetes and cancer: a consensus report. *Diabetes Care* 2010, 33(7):1674–1685. doi: 10.2337/dc10-0666 20587728 PMC2890380

[19] Vigneri P, Frasca F, Sciacca L, Pandini G, Vigneri R: Diabetes and cancer. *Endocr Relat Cancer* 2009, 16(4):1103–1123. doi: 10.1677/ERC-09-0087 19620249

[20] Tsilidis KK, Kasimis JC, Lopez DS, Ntzani EE, Ioannidis JP: Type 2 diabetes and cancer: umbrella review of meta-analyses of observational studies. *Bmj* 2015, 350:g7607. 10.1136/bmj.g7607 25555821

[21] Renehan AG, Zwahlen M, Minder C, O’Dwyer ST, Shalet SM, Egger M: Insulin-like growth factor (IGF)-I, IGF binding protein-3, and cancer risk: systematic review and meta-regression analysis. *Lancet* 2004, 363(9418):1346–1353. doi: 10.1016/S0140-6736(04)16044-3 15110491

[22] Population by region, marital status, sex and year [http://www.statistikdatabasen.scb.se/pxweb/en/ssd/START__BE__BE0101__BE0101A/BefolkningNy/table/tableViewLayout1/].

[23] Population by region, sex, region of birth and year [http://www.statistikdatabasen.scb.se/pxweb/en/ssd/START__BE__BE0101__BE0101E/InrUtrFoddaRegAlKon/table/tableViewLayout1/].

[24] Population by region, age, sex, region of birth and year [http://www.statistikdatabasen.scb.se/pxweb/en/ssd/START__BE__BE0101__BE0101E/InrUtrFoddaRegAlKon/table/tableViewLayout1/].

[25] Population by region, marital status, age, sex and year [http://www.statistikdatabasen.scb.se/pxweb/en/ssd/START__BE__BE0101__BE0101A/BefolkningNy/table/tableViewLayout1/].

[26] Sundquist K, Malmström M, Johansson S-E, Sundquist J: Care Need Index, a useful tool for the distribution of primary health care resources. *Journal of Epidemiology and Community Health* 2003, 57(5):347–352. doi: 10.1136/jech.57.5.347 12700218 PMC1732439

[27] Calderon-Larranaga A, Vetrano DL, Onder G, Gimeno-Feliu LA, Coscollar-Santaliestra C, Carfi A, et al: Assessing and Measuring Chronic Multimorbidity in the Older Population: A Proposal for Its Operationalization. *J Gerontol A Biol Sci Med Sci* 2017, 72(10):1417–1423. doi: 10.1093/gerona/glw233 28003375 PMC5861938

[28] Santiago-Rodríguez EJ, Rivadeneira NA, Torres JM, Sarkar U, Hiatt RA: Socioeconomic status and colorectal cancer screening behaviors in a vulnerable multiethnic population. *Ethn Health* 2022, 27(4):980–996. doi: 10.1080/13557858.2020.1838454 33121258 PMC8081754

[29] Hur HW, Ryu SY, Park J, Choi SW: Relationship between Socioeconomic Status and Prevalent Prostate Cancer in the South Korea. *Asian Pac J Cancer Prev* 2019, 20(10):3137–3144. doi: 10.31557/APJCP.2019.20.10.3137 31653165 PMC6982686

[30] Sano S, Oshima K, Wang Y, MacLauchlan S, Katanasaka Y, Sano M, et al: Tet2-Mediated Clonal Hematopoiesis Accelerates Heart Failure Through a Mechanism Involving the IL-1β/NLRP3 Inflammasome. *J Am Coll Cardiol* 2018, 71(8):875–886. 29471939 10.1016/j.jacc.2017.12.037 PMC5828038

[31] Zalewska-Szewczyk B, Lipiec J, Bodalski J: Late cardiotoxicity of anthracyclines in children with acute leukemia. *Klin Padiatr* 1999, 211(4):356–359. doi: 10.1055/s-2008-1043814 10472576

[32] Von Hoff DD, Layard MW, Basa P, Davis HL Jr., Von Hoff AL, Rozencweig M, et al: Risk factors for doxorubicin-induced congestive heart failure. *Ann Intern Med* 1979, 91(5):710–717. doi: 10.7326/0003-4819-91-5-710 496103

[33] Yan P, Wang Y, Fu T, Liu Y, Zhang ZJ: The association between type 1 and 2 diabetes mellitus and the risk of leukemia: a systematic review and meta-analysis of 18 cohort studies. *Endocr J* 2021, 68(3):281–289. doi: 10.1507/endocrj.EJ20-0138 33087643

[34] Lee SC, Chan JC: Evidence for DNA damage as a biological link between diabetes and cancer. *Chin Med J (Engl)* 2015, 128(11):1543–1548. doi: 10.4103/0366-6999.157693 26021514 PMC4733759

[35] Roderburg C, Loosen SH, Jahn JK, Gänsbacher J, Luedde T, Kostev K, et al: Heart failure is associated with an increased incidence of cancer diagnoses. *ESC Heart Failure* 2021. doi: 10.1002/ehf2.13421 34180146 PMC8497216

[36] Tang F, Gates Kuliszewski M, Carrascal A, Vásquez E: Physical multimorbidity and cancer prevalence in the National Health and Nutrition Examination Survey. *Public Health* 2021, 193:94–100. doi: 10.1016/j.puhe.2021.01.026 33751964

[37] Mattiuzzi C, Lippi G: Current Cancer Epidemiology. *J Epidemiol Glob Health* 2019, 9(4):217–222. doi: 10.2991/jegh.k.191008.001 31854162 PMC7310786

