# Supplementary material for: Associations of heart failure to prevalence of haematologic- and solid malignancies in southern Sweden: A cross-sectional study
Source: PLoS One. 2023 Oct 13;18(10):e0292853. doi: 10.1371/journal.pone.0292853 (PMC10575512; doi:10.1371/journal.pone.0292853)
Supplement: S1 Appendix — Appendix—contains the following: Appendix Table 1. ICD-codes for the diseases included in present study. Appendix Table 2. Different variables in Model A—E. (DOCX) [file pone.0292853.s002.docx]

| Appendix Table 1. ICD-codes for the diseases included in present study. | | | | | | | | | | | | | |  |
| --- | --- | --- | --- | --- | --- | --- | --- | --- | --- | --- | --- | --- | --- | --- |
|  |  |  |  |  |  |  |  |  |  |  |  |  |  |  |
| **Disease** |  |  |  |  |  |  | **ICD-10 codes** | |  |  |  |  |  |  |
| heart failure | |  |  |  |  |  | I50 |  |  |  |  |  |  |  |
| diabetes mellitus including postprocedural hypoinsulinaemia | | | | | | | E10, E11, E13, E14, E891 | | |  |  |  |  |  |
|  |  |  |  |  |  |  |  |  |  |  |  |  |  |  |
| ***Hematological neoplasms*** | | |  |  |  |  |  |  |  |  |  |  |  |  |
| Hodgkin’s lymphoma | | |  |  |  |  | C81 |  |  |  |  |  |  |  |
| follicular lymphoma | |  |  |  |  |  | C82 |  |  |  |  |  |  |  |
| non-follicular lymphoma | | |  |  |  |  | C83 |  |  |  |  |  |  |  |
| mature T/NK - cell lymphomas | | |  |  |  |  | C84 |  |  |  |  |  |  |  |
| other and unspecified types of non-Hodgkin’s lymphoma | | | | | | | C85 |  |  |  |  |  |  |  |
| other specified types of T/NK - cell lymphoma | | | | |  |  | C86 |  |  |  |  |  |  |  |
| malignant immunoproliferative diseases | | | | | |  | C88 |  |  |  |  |  |  |  |
| multiple myeloma and malignant plasma cell neoplasms | | | | | | | C90 |  |  |  |  |  |  |  |
| lymphoid leukaemia | | |  |  |  |  | C91 |  |  |  |  |  |  |  |
| myeloid leukaemia | | | | |  |  | C92 |  |  |  |  |  |  |  |
| monocytic leukaemia | |  |  |  |  |  | C93 |  |  |  |  |  |  |  |
| other leukaemias of specified cell type | | | |  |  |  | C94 |  |  |  |  |  |  |  |
| leukaemia of unspecified cell type | | | |  |  |  | C95 |  |  |  |  |  |  |  |
| other and unspecified malignant neoplasms of lymphoid, | | | | | | | C96 |  |  |  |  |  |  |  |
| hematopoietic and related tissue | | | |  |  |  |  |  |  |  |  |  |  |  |
|  |  |  |  |  |  |  |  |  |  |  |  |  |  |  |
| ***Solid neoplasms*** | |  |  |  |  |  |  |  |  |  |  |  |  |  |
| malignant neoplasms | | |  |  |  |  | C |  |  |  |  |  |  |  |
| carcinoma in situ of digestive organs, skin, breast, genital organs, | | | | | | | D00, D01, D02, D03, D04, D05, D06, D07, D09 | | | | | |  |  |
| middle ear, respiratory system or other unspecified sites | | | | | | |  |  |  |  |  |  |  |  |
|  | | | | | | |  | | | | | | | |
|  | |  |  |  |  |  |  |  |  |  |  |  |  |  |

| Appendix Table 2. Different variables in Model A - E. | |
| --- | --- |
|  |  |
| **Model** | **Variables** |
|  |  |
| Model A | gender, age |
| Model B | gender, age, heart failure |
| Model C | gender, age, heart failure, diabetes mellitus |
| Model D | gender, age, heart failure, heart failure, diabetes mellitus, socioeconomic status |
| Model E | gender, age, heart failure, diabetes mellitus, socioeconomic status, multimorbidity level |
|  |  |
